# A Case Report of Pembrolizumab-Induced Allergic Hepatitis

**DOI:** 10.7759/cureus.64703

**Published:** 2024-07-16

**Authors:** Saho Nakatani, Munehisa Fukushima, Shiro Akahani

**Affiliations:** 1 Otolaryngology and Head and Neck Surgery, Kansai Medical Hospital, Toyonaka, JPN; 2 Otolaryngology and Head and Neck Surgery, Tokyo Women’s Medical University, Tokyo, JPN; 3 Otolaryngology and Head and Neck Surgery, Kansai Rosai Hospital, Amagasaki, JPN

**Keywords:** lymphocyte transformation test, immune-related adverse events (iraes), allergic hepatitis, pembrolizumab, immunotherapy

## Abstract

The immune checkpoint inhibitor pembrolizumab is now considered a first-line treatment for recurrent or metastatic head and neck squamous cell cancer. Pembrolizumab is less toxic than conventional chemotherapy but may result in immune-related adverse events. We report a case in which liver injury occurred just two days after the administration of pembrolizumab plus chemotherapy. A 48-year-old woman achieved a complete response after chemoradiotherapy for cT2N3bM0 squamous cell carcinoma of the oropharynx with multiple lymph node metastases. However, the tumor recurred one year later, and she was started on pembrolizumab plus chemotherapy. On day 3, her alanine aminotransferase and aspartate transaminase concentrations markedly increased. She was initially diagnosed with drug-induced liver injury and all medications were withdrawn. Her liver function recovered within two weeks without intervention. The lymphocyte transformation test was only positive for pembrolizumab. Clinicians should consider pembrolizumab-induced allergic hepatitis as a possible cause of liver injury after excluding liver metastasis and immune-related adverse events.

## Introduction

The first-line treatment for recurrent or metastatic head and neck squamous cell cancer now includes the immune checkpoint inhibitor pembrolizumab, which is a humanized monoclonal antibody against the protein programmed cell death 1 that has lower toxicity than conventional chemotherapy [1-3]. However, patients treated with immunotherapy may experience specific immune-related adverse events that affect various organs. During pembrolizumab treatment, mild or moderate elevation of the liver enzymes is common, and severe hepatotoxicity occurs in 1% to 4% of patients [1]. Liver injury usually occurs at one to three months after two to four cycles of pembrolizumab treatment and often spontaneously recovers without treatment discontinuation [4-6].

Herein, we report a case in which liver injury occurred just two days after the administration of pembrolizumab plus chemotherapy, leading to a diagnosis of pembrolizumab-induced allergic hepatitis based on the result of a lymphocyte transformation test, after excluding other causes. A previous study reported that the most commonly identified cause of liver injury is hepatic metastases rather than pembrolizumab-induced hepatotoxicity [7]. To our knowledge, this is the first report identifying pembrolizumab as the possible cause of drug-induced allergic hepatitis.

## Case presentation

The patient was a 48-year-old woman with no history of drug and food allergies, no smoking history, no relevant medical history, and no family history of cancer. She consulted our institution for right neck swelling and was histologically diagnosed with cT2N3bM0 squamous cell carcinoma of the oropharynx with multiple lymph node metastases. She underwent chemoradiotherapy using 80 mg/m^2^ of cisplatin every three weeks with intensity-modulated radiation therapy to a total dose of 70 Gy, achieving a complete response confirmed by positron emission tomography. However, one year later, the tumor recurred in the right internal jugular chain region with vascular invasions. Therefore, administration of pembrolizumab plus chemotherapy (cisplatin and 5-fluorouracil) was planned for her recurrent unresectable lesion.

**Figure 1:**
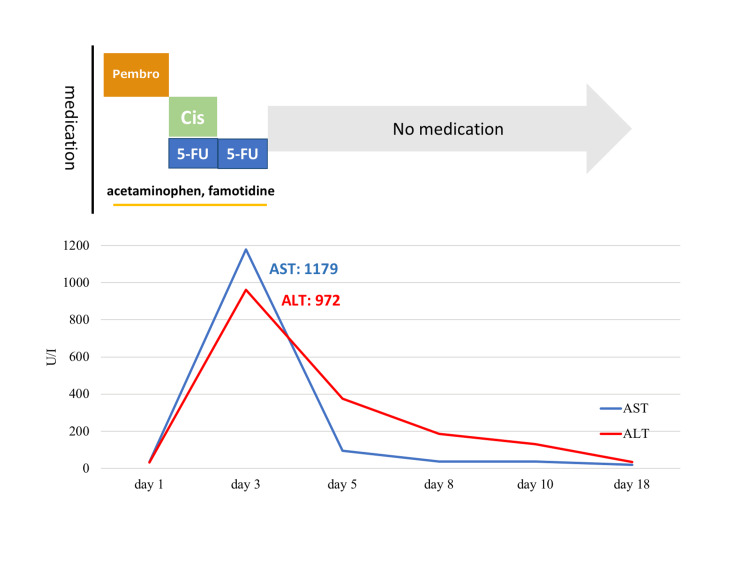
Time course of medications and hepatic enzyme concentrations The ALT and AST concentrations rapidly increased on the third day after administering pembrolizumab plus chemotherapy, followed by a rapid decrease in these concentrations after all medications were discontinued. Pembro: pembrolizumab; Cis: cisplatin; 5-FU: 5-fluorouracil; ALT: alanine aminotransferase; AST: aspartate transaminase; Y-axis: hepatic enzyme concentrations; X-axis: time course

On day 3, after the first dose of pembrolizumab plus chemotherapy, she had marked elevations of her alanine aminotransferase (ALT) and aspartate transaminase (AST) concentrations without subjective symptoms and was classified as having Grade 4 liver injury according to the Common Terminology Criteria for Adverse Events v5.0 (Figure 1).

She was initially suspected to have drug-induced liver injury and all medications, including chemotherapy, were stopped. Her liver function rapidly recovered within two weeks without any specific therapy. From the second round of chemotherapy three weeks after the liver injury, her cancer was treated with cisplatin plus 5-fluorouracil only. There was no recurrence of liver enzyme derangement.

## Discussion

**Figure 2:**
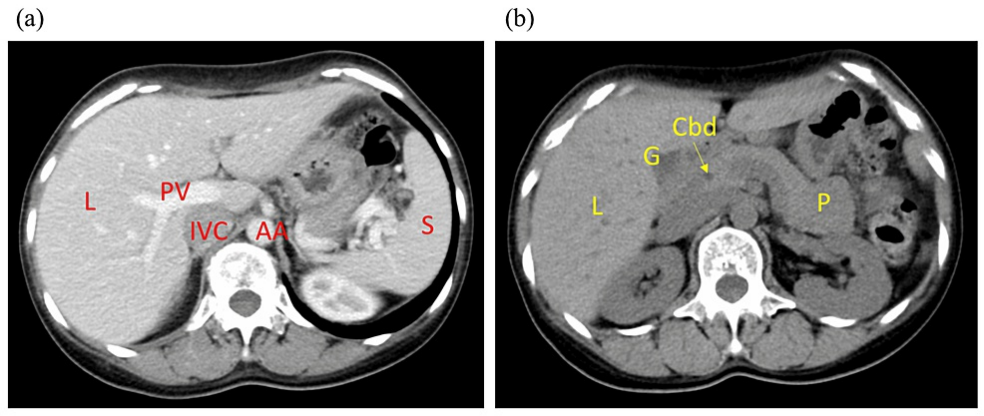
Abdominal computed tomography images (a) Contrast-enhanced image; (b) non-contrast image. Both images show no significant abnormal findings in the liver and biliary system, including the gallbladder and intra- and extrahepatic bile ducts. AA: abdominal aorta; Cbd: common bile duct; G: gallbladder; IVC: inferior vena cava; P: pancreas; PV: portal vein; S: spleen; L: liver

In the present case, we examined other possible causes of liver damage such as viral or autoimmune hepatitis based on abdominal ultrasonography, computed tomography, and laboratory studies. Her abdominal ultrasonography was normal, contrast-enhanced abdominal computed tomography revealed no significant abnormalities (Figure 2), and the virology screen of an autoantibody screen was negative.

Furthermore, the liver enzymes were markedly elevated only two days after starting the treatment regimen including pembrolizumab. Such an immediate reaction to medication strongly suggested the possibility of acute allergic liver injury. In the present case, the five drugs potentially involved in the liver injury were pembrolizumab, cisplatin, 5-fluorouracil, acetaminophen, and famotidine. The lymphocyte transformation test was performed for all medications, and only pembrolizumab was positive with a stimulation index of 2.8. According to the Roussel Uclaf Causality Assessment Method, pembrolizumab was classified as "probable" [8,9]. Thus, we concluded that her liver injury was pembrolizumab-induced allergic hepatitis. Based on these findings, her liver injury was diagnosed as an allergic reaction induced by pembrolizumab.

Previous reports indicate that the adverse events during pembrolizumab treatment are variable and include immune-related adverse events, systemic inflammation, and musculoskeletal problems [10]. Immune-related adverse events can affect multiple organs such as the skin, gastrointestinal tract, endocrine system, lungs, nervous system, musculoskeletal system, and liver [11]. In the present case, the liver injury occurred rapidly after the administration of pembrolizumab, suggesting that this emerging drug can cause acute allergic liver injury. In cases with early increases in liver enzyme concentrations, it is important to consider an allergic drug reaction as a potential cause.

## Conclusions

The immune checkpoint inhibitor pembrolizumab is now considered a first-line treatment for recurrent or metastatic head and neck squamous cell cancer. It is essential to educate clinical staff on the safe and appropriate usage of this recently introduced pharmaceutical agent. Early recognition and management of adverse events are crucial.

To our knowledge, this is the first report to identify pembrolizumab as the cause of drug-induced allergic hepatitis. When a patient develops liver injury during pembrolizumab treatment, clinicians should consider pembrolizumab-induced allergic hepatitis as a possible diagnosis among other causes such as liver metastasis or immune-related adverse events.
